# Effectiveness of electroconvulsive therapy in patients with “less treatment‐resistant” depression by the Maudsley Staging Model

**DOI:** 10.1002/brb3.1654

**Published:** 2020-05-13

**Authors:** Yarong Ma, Robert Rosenheck, Biyu Ye, Ni Fan, Hongbo He

**Affiliations:** ^1^ Affiliated Brain Hospital of Guangzhou Medical University (Guangzhou Huiai Hospital) Guangzhou China; ^2^ Department of Psychiatry Yale School of Medicine New Haven CT USA

**Keywords:** depression symptoms severity, effectiveness, electroconvulsive therapy, treatment‐resistance

## Abstract

**Introduction:**

Electroconvulsive therapy (ECT) is an effective treatment for patients with mood disorders and is most often used for treatment‐resistant cases. This study aimed to examine the effectiveness of ECT in a real‐world treatment sample in a Chinese psychiatric hospital which included both treatment‐resistant and nontreatment‐resistant patients.

**Methods:**

An observational study of symptom outcomes from admission to the time of discharge was conducted with 37 inpatients diagnosed with unipolar or bipolar depression treated with ECT. Symptom severity was assessed with the 17‐item Hamilton Rating Scales for Depression (HRSD‐17) and treatment‐resistance with the Maudsley Staging Model (MSM). Stratifying at the MSM median admission characteristics and symptom change was compared between patients who were treatment‐resistant (*n* = 18) and who were not (*n* = 19). The outcome difference between groups was compared using analyses of covariance adjusted for baseline characteristics including symptom severity, followed by linear regression to identify factors associated symptom improvement in the entire sample.

**Results:**

The sample (*n* = 37) showed moderate treatment‐resistance (MSM = 7.30 ± 1.13) at admission and both groups received 8.3 ± 2 ECT sessions. The treatment‐resistant group had a smaller proportion of bipolar patients and more severe symptoms, but showed no significant difference from the nontreatment‐resistant group in HDRS‐17 scores at the time of discharge (adjusted means = 6.23 ± 1.00 vs. 5.94 ± 0.97, Partial *η* ^2^ = 0.001, *p* = .845). Baseline symptom severity was the strongest correlate of reduction in HDRS‐17 scores (*β* = 0.891, *p* < .001).

**Conclusions:**

Symptom change with ECT in depression did not differ by level of treatment‐resistance but was greatest among those with more severe baseline symptoms. Correlates of ECT effectiveness should be further evaluated in stratified randomized trials.

## Graphical abstract

The reduction of depressive symptoms with ECT does not appear to be affected by overall “treatment‐resistance” but is greatest for those with the most severe symptoms at baseline.

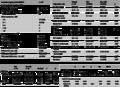

## 1 INTRODUCTION

Electroconvulsive therapy (ECT) is widely believed to be the most efficacious treatment for depression (Mutz et al., 2019) and is used most often when antidepressant medications have failed to yield adequate clinical improvement (Lisanby, 2007), that is, in treatment‐resistant depression (TRD) defined as failure to respond to two or more adequate trials of antidepressant medications (Brown et al., 2019).

In December 2018, the Food and Drug Administration (FDA) reclassified ECT machines as class II medical devices (Barton, 2018) and formally recommend use of ECT for patients with severe depression who are treatment‐resistant or who require a rapid response due to the severity of their psychiatric or medical conditions (Barton, 2018). Most randomized controlled trials of ECT have focused on demonstrating its efficacy in TRD (Kellner & Nordenskjold, 2019), and as a result little is known about its use in on‐TRD cases.

In contrast to the FDA guidelines and community practice in the United States, Chinese guidelines on the use of ECT recommend its relatively flexible use in treating severe unipolar or bipolar depression even before treatment‐resistance is established. These guidelines recommend use of ECT in response to severe symptoms or serious suicidality (Zhou et al., 2017) in view of the fact that, regardless of treatment‐resistance, ECT works more rapidly than medications (Spaans et al., 2015). These differences between guidelines are reflected in dramatically different rates of use with only 0.25% use of ECT in one large study in US (Wilkinson, Agbese, Leslie, & Rosenheck, 2018) as compared to 25.8% in China (Ma, Rosenheck, Fan, & He, 2019).

The US practice of using ECT primarily after other treatments have failed may be justified, if ECT is specifically beneficial only in treatment‐resistant cases. On the other hand, it is possible that ECT is similarly effective in highly symptomatic patients for whom treatment‐resistant has not been demonstrated, even though evidence of such efficacy is currently lacking (Dudleston, 2019; Kellner et al., 2015; Kellner, Popeo, Pasculli, Briggs, & Gamss, 2012).

In view of the typically more restrictive use of ECT in the United States, data from Chinese hospitals in which ECT is used to treat both patients with demonstrated treatment‐resistance and those with severe symptoms alone may be useful in comparing the effectiveness of ECT in both kinds of patients. The present study uses data on patients treated with ECT in a large psychiatric hospital in Guangzhou, China to compare baseline characteristics and changes in symptom severity between admission and discharge among those with treatment‐resistance, as demonstrated by the Maudsley Staging Model (Fekadu et al., 2009) and those with severe symptoms but without demonstrated treatment‐resistance.

## 2 METHODS

### 2.1 Study design and participants

This observational, quasi‐experimental study compared outcomes from the time of admission to the time of discharge among patients diagnosed with unipolar or bipolar depression during a clinically depressive phase who received ECT during a hospitalization at the Guangzhou Huiai Hospital.

The sample included 37 patients from the project examined "risk factors for a prolonged length of stay and readmission for patients with mental illness" (He et al., 2015) in which the data on ECT effectiveness have not been analyzed or published before. The study was conducted at Guangzhou Huiai Hospital, the largest psychiatric hospital in Guangdong Province, China, between February 2012 and October 2013. The clinical diagnoses of depression were confirmed by two experienced psychiatrists using ICD‐10 criteria.

Treatment‐resistance was measured by the Maudsley Staging Model (MSM, 2009) (Fekadu et al., 2009), which summarizes the level of TRD in a single score, varying between 3 and 15, and defines three ordinal categories of treatment‐resistance: mild (scores = 3–6), moderate (scores = 7–10), and severe (scores = 11–15) (Ruhe, van Rooijen, Spijker, Peeters, & Schene, 2012). Since most patients in this sample received ECT for rapid response or suicidality and not on the basis of the number of treatment failures, we used the number of types of antidepressants the patient had ever taken as the indicator of antidepressant failure and hence treatment‐resistance in the MSM. Stratified by an MSM median of 7, 18 patients (49%) were identified as having treatment‐resistant depression (TRD) while 19 (51%) did not (non‐TRD).

### 2.2 ECT procedures

ECT treatments at Guangzhou Huiai Hospital are scheduled three times a week using the Mecta Spectrum 5000 device (Mecta Corp). After pre‐oxygenation with 100% oxygen, propofol anesthesia (or etomidate if clinically indicated) is combined with the muscle relaxant suxamethonium, unless a change was required for clinical reasons in specific cases. Electrode placement is the standard bitemporal placement. After confirmation of complete muscle relaxation (cessation of pedal muscle fasciculation), a seizure is induced by giving patients constant‐current brief pulse stimuli (0.5 ms pulse width, increased to 1.0 ms if clinically indicated). The treatment dose is calculated as age multiplied by 5 (mC) in the first ECT session (increased to 1.5 or 2 times this figure if necessary to induce a seizure). The stimulus parameters remain the same during the entire ECT treatment course (targeted as 6–12 sessions); unless change is required for clinical reasons. ECT treatments are administered three times weekly until completion of the course of ECT treatment, the duration of which is determined by each patient's treatment team on clinical grounds, with the goal of achieving rapid substantial improvement. EEG monitoring is used to identify induction of seizures.

### 2.3 Measures

Demographics included gender and age. Clinical characteristics included age at first onset, length of stay, duration of the current episode, suicidality (none, ideation, or attempt once), and number of ECT treatments.

The Hamilton Rating Scale for Depression‐17 items (HRSD‐17) (Hamilton, 1967) was used to assess the severity of depression symptoms and was administered by the treating clinician at admission and discharge. Ten HRSD items use a 5‐point Likert scale response format, ranging from 0 to 4 (0 = none, 1 = mild, 2 = moderate, 3 = severe, and 4 = extremely severe) and seven use a three‐category ordinal scale, ranging from 0 to 2 (0 = none, 1 = mild to moderate, and 2 = severe). The total score of the HRSD‐17 reflects the severity of depression. Using the principal factor method (Shafer, 2006), four subscales of the HRSD‐17 have been identified: anxiety, depression, insomnia, and somatic. The reliability and validity of the Chinese version of HRSD‐17 has been confirmed (Zheng et al., 1988) and it has been widely used both clinically and in clinical trials. The primary outcome measure in this study was the pre‐ to post‐ECT change in HDRS‐17 scores measured as a raw value change and as a percentage change. The secondary outcome measures were response and remission, defined, respectively, as a 50% reduction of the HRSD‐17 and an HRSD‐17 score ≤ 7 (de Zwart, Jeronimus, & de Jonge, 2019).

### 2.4 Statistical analysis

After the descriptive statistics based on the MSM for the full sample, the analysis proceeded in three stages. First, a comparison between TRD and non‐TRD groups at admission was performed using chi‐square tests for categorical variables, *t* test for the continuous variables, and the Mann–Whitney test for non‐normally distributed variables. Given the exploratory nature of our study, the significant differences for each test were established at *p* < .05, 2‐tailed.

Second, analysis of covariate (ANCOVA) was used to compare differences in HDRS‐17 scores between TRD and non‐TRD groups on the HRSD‐17 and its subscales at the time of hospital discharge controlling for measures that were significantly different between groups at baseline. Least square means adjusted for baseline differences were computed and effect size differences were calculated using *η* ^2^ (Richardson, 2011), from the ANCOVA. Eta squared is the proportion of the total variance that is attributed to an effect.

Finally, stepwise linear regression was used to evaluate predictors of the decrease in the HRSD‐17 by value change and by percentage change. Independent variables in the model included demographics, clinical characteristics, MSM score, and baseline HRSD‐17 score. Statistical analyses were performed using JASP 0.11.1 for Windows (An open‐source project supported by the University of Amsterdam).

### 2.5 Ethical statement

Not applicable.

## 3 RESULTS

A total of 37 patients were evaluated with the Maudsley Staging Model showing a moderate average score (MSM = 7.30 ± 1.13) for treatment‐resistance. All of the patients were in an acute episode stage, and most patients (78%) had been prescribed at least two different antidepressant medications. During the current episode, all patients received some augmentation whether with antipsychotics, mood stabilizers, or benzodiazepines. A wide range of depression symptom severity was observed ranging from among mild (8%), to moderate (24%), to severe (68%). Few patients (5%) had received ECT, prior to the current hospitalization (Table 1).

**TABLE 1:** Maudsley staging model assessment descriptive statistics for full sample

Maudsley staging method (MSM)	*n* = 37
Current episode duration, *n* (%)	
Acute (<12 months)	37 (100%)
Subacute (12–24 months)	0
Chronic (>2 years)	0
Failed treatments, *n* (%)^a^	
1–2	29 (78%)
3–4	8 (22%)
5–6	0
7–10	0
>10	0
Depression severity, *n* (%)^b^	
Mild	3 (8%)
Moderate	9 (24%)
Severe without psychosis	9 (24%)
Severe with psychosis	16 (43%)
Augmentation, yes, *n* (%)	37 (100%)
Previous ECT, yes, *n* (%)	2 (5%)
MSM score, Mean ± *SD*	7.30 ± 1.13
TRD cases (MSM score > 7), *n* (%)^c^	18 (49%)

Note

Augmentation, prescribed with psychotics, mood stabilizers, or benzodiazepines.

^a^ The number of types of antidepressants the patient had ever taken as the indicator of antidepressants failure.

^b^ Depression severity was classified by the total score of Hamilton Rating Scares for Depression‐17 items (mild 8 ~ 17, moderate 18 ~ 26, severe 27 or more), and those with psychosis were severe cases according to ICD‐10.

^c^ Treatment‐resistant depression (TRD) versus Non‐TRD cases were stratified by the MSM median (MSM = 7).

The comparison of TRD and non‐TRD patients showed the TRD group to have a significantly a greater percentage diagnosed with unipolar depression, and more severe depressive symptoms at the time of admission (31.0 ± 4.1 vs. 23.4 ± 7.9, *p* < .001). The groups received virtually identical numbers of ECT treatments at 8.2 (Table 2). Before adjusting for baseline characteristics including depressive symptoms, the TRD group achieved a greater unadjusted decrease in HRSD‐17 scores value (24.7 ± 4.9 vs. 17.5 ± 8.6, *p* = .004) but no greater percentage decline from baseline (*p* > .05). Unadjusted comparison showed 95% of the sample responded to the treatment (50% symptom improvement) and 65% achieved remission at discharge with no statistically significant difference between the groups (Table 2).

**TABLE 2:** Demographics, and clinical characteristics by TRD versus no‐TRD group

*n* (%), Mean ± *SD*	Overall *n* = 37	TRD 18 (49%)	Non‐TRD 19 (51%)	*p*
Gender, female	18 (49%)	11 (61%)	7 (39%)	.140^a^
Age, years	26.8 ± 7.3	27.2 ± 8.7	26.5 ± 5.8	.794^b^
First onset age, years	23.1 ± 8.0	23.2 ± 8.7	22.9 ± 7.4	.903^b^
Current episode duration, month	3.0 ± 2.6	2.7 ± 2.4	3.4 ± 2.8	.259^c^
Length of stay, days	54.3 ± 31.4	62.8 ± 37.6	46.3 ± 22.2	.186^c^
Bipolar % (*n*)	19 (51%)	6 (32%)	13 (68%)	.033^a^
Suicidality				
None	13 (35%)	6 (33%)	7 (37%)	.257^a^
Ideation	12 (32%)	4 (22%)	8 (42%)	
Suicide attempt	12 (32%)	8 (44%)	4 (21%)	
ECT sessions	8.3 ± 2.2	8.3 ± 2.3	8.3 ± 2.3	.925^c^
HRSD‐17 at admission	27.1 ± 7.3	31.0 ± 4.1	23.4 ± 7.9	.001^b^
HRSD‐17 at discharge	6.1 ± 3.6	6.3 ± 2.9	5.9 ± 4.2	.750^b^
HRSD‐17 decrease (value)	21.0 ± 7.9	24.7 ± 4.9	17.5 ± 8.6	.004^b^
HRSD‐17 decrease (percentage)	0.762±0.174	0.798±0.099	0.727±0.221	.220^b^
Responders	35 (95%)	18 (100%)	17 (90%)	.157^a^
Remitters	24 (65%)	13 (72%)	11 (58%)	.362^a^

Note

Treatment‐resistant depression (TRD) versus no‐TRD cases were stratified by the MSM median (MSM = 7). And differences were tested by using the chi‐square test^a^, Student *t* test^b^, and Mann–Whitney test^c^.

Responders: at least 50% reduction of the HRSD‐17. Remitters: an HRSD‐17 score ≤ 7.

Abbreviations: HRSD‐17, Hamilton Rating Scares for Depression‐17 items; ECT, Electroconvulsive therapy.

ANCOVA which did adjust for baseline symptoms showed no significant differences between the groups on depression symptoms at discharge, on the HRSD‐17 total score, or on any subscale (Partial *η* ^2^ < 0.1, *p* > .05) (Table 3).

**TABLE 3:** ANCOVA of HRSD‐17 at discharge by TRD versus Non‐TRD (least square mean ± *SE*)

	TRD *n* = 18	Non‐TRD *n* = 19	^a^Partial *η* ^2^	*F*	*p* ^b^
HRSD‐17					
Anxiety	2.14 ± 0.54	3.02 ± 0.52	0.035	1.197	.300
Depression	2.61 ± 0.50	2.00 ± 0.49	0.019	0.640	.429
Insomnia	0.55 ± 0.20	0.43 ± 0.19	0.005	0.172	.681
Somatic	0.64 ± 0.27	0.76 ± 0.26	0.003	0.095	.760
Total	6.23 ± 1.00	5.94 ± 0.97	0.001	0.035	.854

Abbreviations: ANCOVA, analysis of covariate; HRSD‐17, Hamilton Rating Scares for Depression‐17 items; TRD, treatment‐resistant depression.

^a^ Partial *η* ^2^ is the proportion of the total variance that is attributed to an effect.

^b^ Adjusted for baseline HRSD‐17 and the variable bipolar, *df* (TRD) = 1, *df* (error) = 33.

In contrast, the linear regression model indicated that HRSD‐17 decrease was significantly associated with baseline HRSD‐17, in value (*β* = 0.891, *p* < .001) and percentage change (*β* = 0.371, *p* = .024), with MSM score and classification not included in the stepwise models (Table 4).

**TABLE 4:** Stepwise Linear Regression for HRSD‐17 outcomes

	*B*	*SE*	*β*	Sig.	95% CI	
Model for decrease value						
(Intercept)	−4.763	2.299		.046	−9.430	−0.096
HRSD‐17 at baseline	0.951	0.082	0.891	<.001	0.785	1.118
Model for decrease percentage						
(Intercept)	0.524	0.104		<.001	0.313	0.735
HRSD‐17 at baseline	0.009	0.004	0.371	.024	0.001	0.016

Abbreviations: CI, confidence interval; HRSD‐17, Hamilton Rating Scares for Depression‐17 items.

## 4 DISCUSSION

This observational study found no significant differences in the adjusted magnitude of reduction of depressive symptoms after ECT in more and less treatment‐resistant patients diagnosed with depressive mood disorders. More severe baseline depression symptoms predicted a greater benefit from ECT, reflecting greater regression to the mean, to some degree. Thus, in this study change in depressive symptoms following ECT did not significantly differ by treatment‐resistance as measured by the MSM raising the possibility, worthy of experimental evaluation, that ECT could be as effective in non‐TRD patients as it is in those with TRD.

van Diermen et al. (2018) also investigated the potential role of the (MSM) in the prediction of ECT outcome and found that of 65 patients who received ECT for a major depressive episode higher symptom levels and greater duration of illness were associated with greater response, but not past treatment failures, the usual way of identifying TRD.

Despite the fact that the FDA mentioned “symptom severity” as well as “treatment‐resistance” (Barton, 2018), as criteria for using ECT, most ECT in western countries is used as a last resort for patients with several pharmacological treatment failures (Dudleston, 2019; McDonald, Weiner, Fochtmann, & McCall, 2016). The underuse of ECT in western countries was recently reviewed (Read, Cunliffe, Jauhar, & McLoughlin, 2019; Sackeim, 2017; Slade, Jahn, Regenold, & Case, 2017), and it was suggested that its underuse may reflect its image as a symbol of coercion, repression or stigma and may contribute to denying some of the most seriously ill depressed patients one of the most effective treatments for their condition (Read et al., 2019). This perception may thus impede its use, thereby denying some patients to access to an effective treatment (Gazdag & Ungvari, 2019). As Keith Dudleston recently suggested, many inpatients with severe depression, who remain withdrawn and unresponsive, might improve substantially with ECT (Dudleston, 2019).

Kellner et al. (2012) identified three factors that the view as predicting benefit from ECT: severity of depressive symptoms, heritability of depression, and episodic nature of the depression and used these characteristics as the basis for an “ECT Appropriateness Scale (EAS).” The severity of depressive symptoms in the current episode appears, from the present study, to be the most important factor of those included in the EAS. This study is distinctive in evaluating ECT in less treatment‐resistant and less severely depressed patients who do not typically receive ECT or enter into ECT trials. It may be the first to empirically provide data supporting the use of ECT in patients without TRD as suggested by the EAS criteria recommend by Kellner et al.

### 4.1 Limitations

Several methodological limitations of the current study must be acknowledged. First, the lack of randomized controls who did not receive ECT makes it difficult to unambiguously attribute symptom reductions to ECT since they could reflect similar levels of regression to the mean. Second, sample sizes were small; the data were from a single medical center; and all subjects were inpatients, thus limiting the generalizability of our findings. Third, some parameters related to ECT treatment (e.g., seizure quality, EEG seizure duration) and concomitant medication use were not available in the data set and could not be included in the analysis. In addition, since the patients reported in this study were substantially younger than those typically treated with ECT in Western countries, where average ages typically exceed 65 (Brus et al., 2017), the generalizability of our findings to other populations is unknown.

## 5 CONCLUSION

Despite these limitations, this study of patients identified in a hospital where ECT was far more commonly used than in most US hospitals suggests that ECT may be effective in patients with serious symptoms but who do not formally qualify as treatment resistant. These data deserve replication in randomized clinical trials and, if confirmed, further studies would be needed to identify the specific level of symptom severity level at which ECT becomes significantly beneficial.

## CONFLICT OF INTEREST

The authors declare no conflicts of interest.

## AUTHOR CONTRIBUTION

HH, RR, and NF contributed to the conception and design of the work. BY and YM contributed to the collection, analysis, and interpretation of data. YM drafted the manuscript revised by RR and HH. All authors provided the approval for publication of the manuscript.

## Data Availability

The data that support the findings of this study are available from the corresponding author upon reasonable request.
